# Hydraulic drive framework on habitat suitability enhances movement bias of brown trout in stream networks

**DOI:** 10.1038/s41598-025-00216-x

**Published:** 2025-05-14

**Authors:** Francesca Padoan, Giulio Calvani, Giovanni De Cesare, Paolo Perona

**Affiliations:** Platform of Hydraulic Constructions (PL-LCH), ENAC - IIC, École Polytechnique Fédérale de Lausanne (EPFL), Lausanne, Switzerland

**Keywords:** Restoration ecology, Hydrology

## Abstract

Freshwater ecosystems face increasing threats from human activities and climate change, thus requiring efforts to reduce impacts and prevent further deterioration. Restoration projects for riverine environments often rely on hydraulic, habitat, and metapopulation models, which depend on their accuracy in capturing the interactions among the different elements. In this regard, a critical challenge is represented by the definition of reliable relationships between fish population dynamics among river reaches in a catchment and habitat characteristics. This study presents an efficient, eco-hydraulically based framework to model the movement bias of brown trout within a river network. The proposed framework integrates the effects of hydraulics and habitat characteristics in terms of the Habitat Suitability Index for various trout age classes. Novel relationships linking fish dynamics to habitat quality are employed to correct the movement bias of fish based on the upstream and downstream habitat conditions. The corrected bias is used to compute the population dynamics at the catchment scale and tested against different flow regimes. Accordingly, the impact of habitat condition changes on the population dynamics is highlighted. The framework simplicity makes it suitable for future integration into existing habitat and metapopulation models, thus offering a practical tool for enhancing riverine restoration planning.

## Introduction

Human activities and climate change are increasingly threatening aquatic biodiversity and freshwater ecosystems^1^ due, for example, to habitat degradation, flow modifications, hydro-power exploitation (with flow diversion)^2^, barriers, invasive species, and diseases^3^. To further limit or reduce the deterioration of fluvial systems, governments and stakeholders can implement river restoration and rehabilitation projects. However, quantitative approaches to aquatic biology and ecohydraulic dynamics are still poorly implemented. For the specific case of Switzerland, several restoration and mitigation projects are to be implemented in the coming decades. According to the revised Swiss *Water Protection Act*, 4000 km of rivers, streams, and lake shores have to be restored by 2090^4^.

**Fig. 1:**
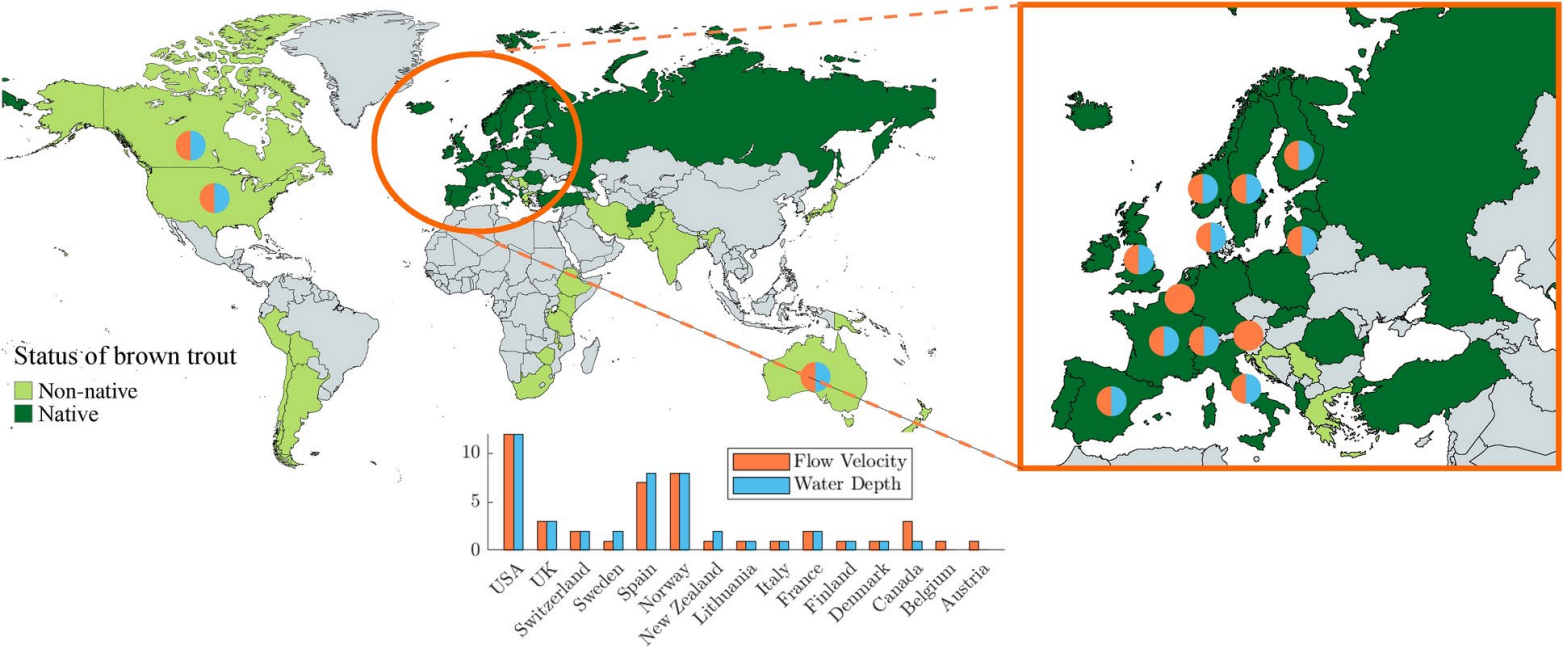
The distribution of native and non-native brown trout worldwide with a histogram showing the data availability in terms of flow velocity (orange) and water depth (cyan) used by Paodan et al.^6^ to build the Habitat Suitability Curves. Map generated via www.mapchart.net using the data of Muhlfeld et al.^5^.

In a broader context, the European Union has recently approved the *Nature Restoration Law*, which addresses the environmental degradation caused by human activities. The EU regulation requires Member States to restore at least 20% of land and sea areas by 2030 and to extend the restoration measures to all the habitats with the ultimate goals of recovering freshwater biodiversity, stopping further loss, improving ecosystems, and enhancing the resilience of nature against climate change by 2050^7,8^. Similar strategies are put in place at the worldwide scale.

A good project achievement depends on the availability of appropriate criteria to identify the most endangered areas and to evaluate the effects on the inhabiting organisms. In the recent decades, increasing attention has been paid to the development and validation of reliable methodologies for scientists and experts to identify the best solutions aiming at biodiversity conservation^9,10^. Despite these advances, decision-making process still relies mainly on local data, and comparisons between case studies from different catchments are seldom available. The after-restoration monitoring process is not yet well-defined^11^, particularly as far as the quantitative assessment of alluvial river attributes is concerned^12^. Furthermore, the development of precise tools capable of predicting the short- and long-term effects of river restoration measures on fish population dynamics and habitat quality is still in its infancy. In this regard, particular focus on threatened and endangered species should be paid, since the effects of in-channel reworking directly influence freshwater organisms. Among potentially-threatened organisms, brown trout (*Salmo trutta L.*) is a classic target species to evaluate the performance of restoration projects, due to its socio-economically importance among freshwater fish^13^. As far as Switzerland is concerned, brown trout is classified as *highly threatened*^14^, whereas the species is labeled as *least concern* at the world scale by the International Union for Conservation of Nature’s Red List of Threatened Species^15^.

When addressing ecosystems and biodiversity loss, it is crucial to consider freshwater habitats and organisms as integrated, interconnected systems. The same comprehensive approach should be taken into account in the design of restoration measures aiming at the protection of target species. To optimize project outcomes, it is essential to simultaneously address both the ecological and environmental requirements of the target species and the whole ecosystem. Consequently, it is important to integrate ecological and hydrological needs with habitat quality, the latter usually evaluated according to models available in the literature^16^. In this context, the most common species-specific tool is the Habitat Suitability Index (HSI), which quantifies habitat conditions at different spatial scales, ranging from small areas (i.e., patch scale, microhabitat) to whole river reaches^17,18^. The HSI may consider different environmental factors such as water depth, flow velocity, and temperature, and takes values from 0 to 1 indicating the likelihood that a target species will live and survive in that habitat. When dealing with habitat modeling, the tools can be further extended by including the migration dynamics of fish between different sites (i.e., reaches) in a river network. The so-called *metapopulation models*, which are used to predict population dynamics, may account for several parameters to determine the dispersion rate and direction of movement between places. Particularly, the direction can be influenced by species-related factors and by network forcings^19–21^. Taking into account, or not, these factors, leads to the development of biased/unbiased metapopulation models^22^.

In this work, starting from a global collection of data on habitat characteristics for brown trout^6^ (Figure 1), we propose an eco-hydraulically based framework which improves the modeling of movement bias of brown trout. In this work, we apply the proposed framework to assess the movement bias at the reach scale within a river network, according to local hydrodynamic conditions and habitat quality. Nevertheless, the tool can be readily extended to different spatial scales, and integrated into existing habitat and metapopulation models, due to its computationally efficient reliability. As a result, the proposed framework ensures the proper quantification of the HSI for researchers, practitioners, and engineers to design restoration and rehabilitation measures. Additionally, the tool may be useful for citizens and politicians to explore improving solutions to live and interact with fluvial networks.

## Methods

**Fig. 2:**
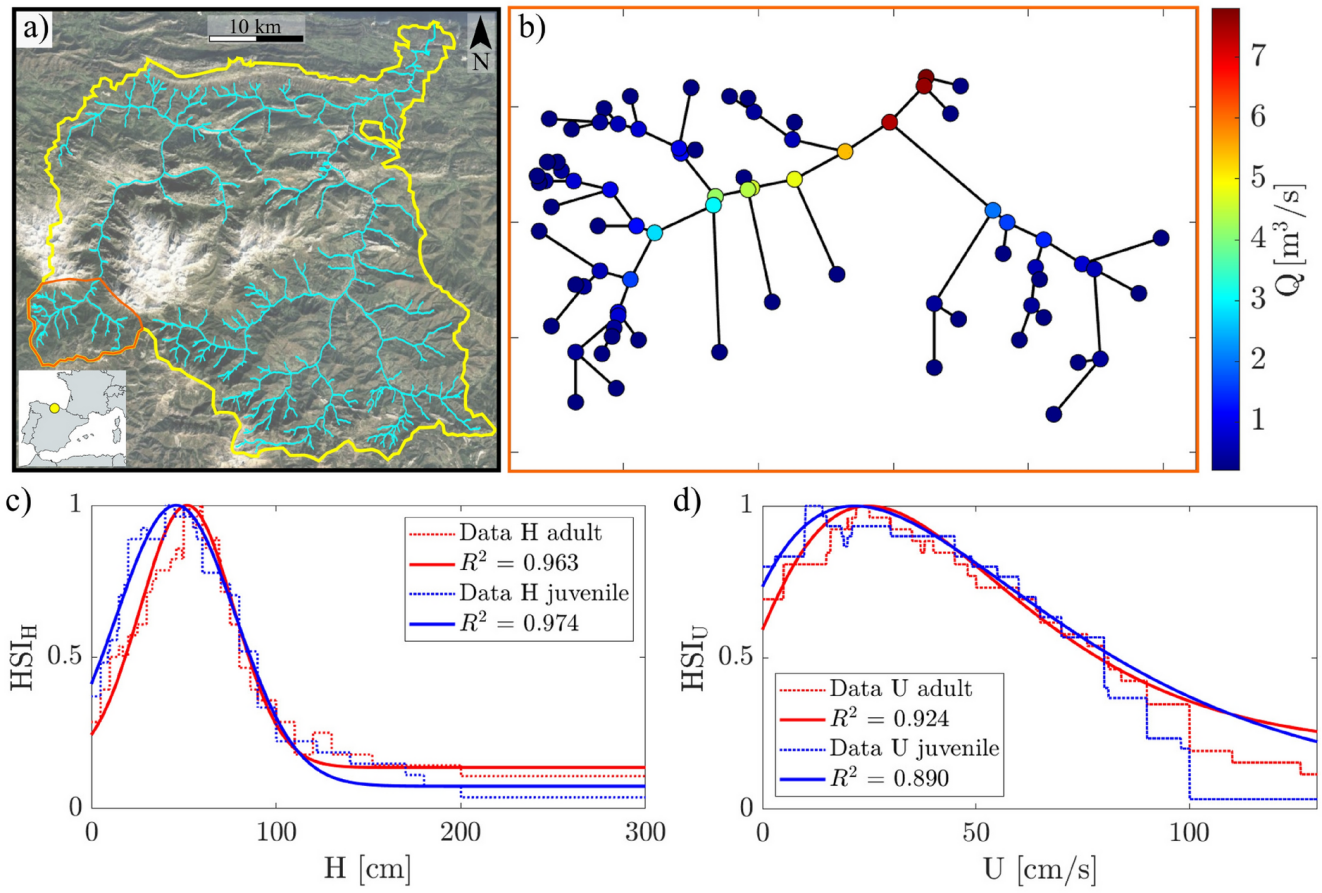
(**a**) Satellite image of the Cares catchment, the orange shape delimits the study case of this work; (**b**) the modeled river network showing the values of flow discharge at each node for an upstream value $Q_0 = 0.2$ m$^3$ s$^{-1}$; (**c**) Fit of a Gaussian distribution of the Habitat Suitability Curves for water depth (dashed lines) for adults (red solid line) and juveniles (blue solid line); (**d**) Fit of a Gamma distribution of the Habitat Suitability Curves for flow velocity (dashed lines) for adults (red solid line) and juveniles (blue solid line).

We refer to the metapopulation model of the river network of Gonz$\acute{\text {a}}$lez-Ferreras et al.^22^ in order to develop an improved movement-bias framework that incorporates the Habitat Suitability Curves, HSC (i.e., mathematical functions defining the relationships between hydraulic properties and HSI). As a result, the effects of habitat quality on the dynamics of trout populations can be easily assessed at the reach scale based on a straightforward computational procedure. Firstly, we calculate the reach-averaged hydraulic characteristics of the whole river network by adopting a simple monodimensional approach. Then, a reanalysis of the data collected by Padoan et al.^6^ is performed to find mathematical relationships between hydraulic variables and the Habitat Suitability Index. Lastly, a novel relationship is proposed to define the fish movement bias by scaling the constant value defined by Gonz$\acute{\text {a}}$lez-Ferreras et al.^22^ as a function of hydrodynamic and habitat conditions of each reach. For comparison with results available in the literature, we apply the proposed methodology to the upstream part of the Cares river network (northern Spain^22^, Figure 2a). In the following, each step of the analysis is explained in detail.

### Implementation of 1D hydrodynamic model

We use the river network model by Gonz$\acute{\text {a}}$lez-Ferreras et al.^22^, where river junctions and reaches are essentially schematized by points (i.e., nodes) and straight segments, respectively (i.e., a graph, see Figure 2b). Here we recall that in the river network, by definition, the most upstream nodes (named source nodes hereafter) have no further upstream links, and the most downstream node (i.e., the outlet of the catchment) has only upstream links. All other nodes in the river network have two upstream links and one downstream^23^. The flow discharge at the source nodes can be retrieved according to the hydrological modeling, the duration of the precipitation, and the catchment area. For simplicity, and without any loss of generality, we use a single discharge value, $Q_0$, for all the source nodes. For more realistic analysis, this value can be made temporally and/or spatially variable to reproduce effects such as seasonality or precipitations. We calculate the flow discharge in the downstream part of the river network by simple continuity principles. Notice that the index 0 refers to the lowest order of the stream. Consequently, for continuity reasons, the flow discharge in any downstream river reach is the sum of the flow rates in the two upstream branches (Figure 2b).

To compute the values of the hydraulic parameters (i.e., water depth and flow velocity), river width, reach-averaged bed slope, and hydraulic roughness (Manning roughness coefficient), must be known at each node (i.e., reach) of the river network. If not available (e.g., for ungauged basins), these can be assumed from the properties of the hydrogeomorphological network statistics and the well-known regime theory empirical relationships^23^. In this work, we set the values of Manning roughness coefficient and slope as fixed depending on the Strahler order (a measure of the stream branching complexity, SO) of the reach. For the sake of simplicity and computational efficiency, cross-sections are assumed rectangular, and normal flow conditions are imposed throughout the entire river network. Accordingly, the calculation of water depth and mean flow velocity in each river reach can be performed by means of the Manning equation

1 $$\begin{aligned} Q = \frac{1}{n}\, \Omega \ R_h^{\frac{2}{3}}\, \sqrt{S} \end{aligned}$$

where $\Omega = B H$ is the cross-sectional wetted area with *B* identifying river width and *H* water depth, *S* is the reach-averaged bed slope, *n* is the Manning roughness coefficient, and $R_h = \frac{B H}{B + 2 H}$ is the hydraulic radius. Eq. (1) can be solved in terms of the mean water depth, *H*, by iterative calculation. Then, the mean flow velocity, *U*, can be calculated as $U = Q / (B H)$. This approach can easily be improved for applicative purposes, using classical hydraulic engineering methods that may account for composite cross section properties^24^.

### Habitat suitability curves and habitat conditions

We take into account the global datasets retrieved by Padoan et al.^6^ (Figure 1) based on several works in the literature. This database includes the river habitat conditions of different brown trout age classes in terms of hydraulic variables (i.e., water depth and mean flow velocity), temperature and mean sediment grain size. Specifically, we use only data on juveniles and adults, and the hydraulic variables of water depth and mean flow velocity (Figure 2c,d). We aim to find mathematical relationships by fitting these datasets. Furthermore, the fitting formulations can be later used for calculations in simulation tools (i.e., habitat and metapopulation models).

As a result, the fitting relationships represent the Habitat Suitability Indices HSI$_H$ and HSI$_U$, as a function of the water depth and the mean flow velocity, respectively. The values of HSI$_H$ and HSI$_U$ can be computed for each age class at each node of the river network according to the flow discharge value and the corresponding water depth and mean flow velocity (Eq. (1)). The two values are then used to calculate the compound Habitat Suitability Index, HSI$_C$, at each node of the river network. In this work, the HSI$_C$ is defined as the geometric mean between the HSI$_H$ and the HSI$_U$, as

2 $$\begin{aligned} HSI_C = \sqrt{HSI_H \cdot HSI_U} \end{aligned}$$

To average the HSI for *U* and *H* we use the geometric mean since it is the most meaningful and also commonly used method to calculate the HSI$_C$ (^25–27^, among others).

### Definition of the weighted bias

The probability of individuals moving from one node to another is related to a “bias” parameter defining the direction of movement. According to the age class, the sign of the bias parameter, which is the difference between the probabilities for an individual to move upstream or downstream, identifies whether the fish prefer to move downstream (negative sign, adult fish that look for more suitable areas and satisfy their food requirements^21,28^) or upstream (positive sign, juvenile fish that try to avoid competition and predators^19,20,29^). Herein, we extend the approach of the constant bias^22^ by including the dependence on the habitat conditions and the gain/loss of habitat suitability when moving between nodes. In particular, we compute the bias for the two upstream nodes through a logistic-type function depending on the ratio between the HSI$_C$ of the adjacent upstream nodes, *j*, and the current node, *i*. Then, the bias for the downstream movement can be computed starting from the two upstream ones. We shall remark further about the use of the logistic function in the discussion section.

We use the Verhulst logistic function^30^ varying between a minimum value, $b_\text {min}$, of the bias, when the adjacent node has a very low HSI (in the limit of HSI$_{c,j}$/HSI$_{c,i}\rightarrow 0$), and a maximum value, $b_\text {max}$, when the HSI-ratio becomes very large (in the limit of HSI$_{c,j}$/HSI$_{c,i}\rightarrow \infty$). When the HSI-ratio is equal to 1, the bias, *b*, becomes equal to the value proposed by Gonz$\acute{\text {a}}$lez-Ferreras et al.^22^ ($b_0$=0.2). Specifically, $b_0$ is equal to -0.1 for adults and 0.1 for juveniles, to account for the presence of the two upstream nodes. The relationship for the bias, $b_j$, towards the upstream node *j*, reads:

3 $$\begin{aligned} b_j = \, \text {sign}(b_0) \, \frac{b_\text {max}}{1+\left( \frac{b_\text {max}}{b_\text {min}}-1\right) \, k^{\left( \frac{HSI_{c,j}}{HSI_{c,i}}\right) ^a}} \end{aligned}$$

where sign($b_0$) depends on the age class, *a* is a coefficient that models the sensitivity of fish to the change in habitat conditions between nodes and is taken equal to 2 for simplicity, and *k* is equal to:

4 $$\begin{aligned} k = \frac{b_\text {max} \, - \, |b_0|}{b_\text {max} \, - \, b_\text {min}} \ \frac{b_\text {min}}{|b_0|} \end{aligned}$$

For the sake of clarity, in this work we use $b_\text {min}=|b_0|/2$ and $b_\text {max}=2|b_0|$. The movement probability, $P^{\text {up}}_j$ towards the upstream node, *j*, can be computed from the bias, $b_j$ as

5 $$\begin{aligned} P^{\text {up}}_j = \frac{1-b_j}{4} \end{aligned}$$

and the movement probabilities among nodes must sum to 1, according to the balance equation. Consequently, the bias towards the downstream node can be calculated as the minus-sum of the bias towards the two upstream nodes. Accordingly, the probability of movement towards the downstream node reads:

6 $$\begin{aligned} P^{\text {dn}} = 1 - \sum _{j=1}^2 \ P^{\text {up}}_j \end{aligned}$$

To assess changes in habitat conditions and movement bias, we test different scenarios of upstream flow discharge, $Q_0$, and determine the habitat conditions in terms of the compound index, HSI$_C$.

## Results

Several functions have been tested using the *curve fitter* toolbox in MATLAB to find the best correlation between data of HSI and modeled values. As a result, it turns out that the Gaussian and Gamma functions have the best performance in terms of correlation coefficient, $R^2$. Specifically, the Gaussian is the best function to fit the curves for water depth, *H*, for both age classes (Figure 2c), while the Gamma is more suitable for water velocity, *U*, always for both age classes (Figure 2d). Specifically, the HSI functions for adults (superscript *A*) read:

7 $$\begin{aligned} HSI_H^A= 0.1357 + 0.8643 e^{-\left( \frac{H -51.78}{35.87}\right) ^2} \end{aligned}$$

and

8 $$\begin{aligned} HSI_U^A= 0.1+0.9 \left( \frac{e}{1.421}\right) ^{1.421} \left( \frac{U+12.08}{25.71}\right) ^{1.421} \ e^{-\frac{U+12.08}{25.71}} \end{aligned}$$

for water depth (subscript *H*) and flow velocity (subscript *U*), respectively. For juvenile (superscript *J*), the fitting relationships read

9 $$\begin{aligned} HSI_H^{J}= 0.074 + 0.926 e^{-\left( \frac{H -45.85}{45.62}\right) ^2} \end{aligned}$$

and

10 $$\begin{aligned} HSI_U^J= 0.06047+0.93953 \left( \frac{e}{1.113}\right) ^{1.113} \left( \frac{U+15.1}{32.95}\right) ^{1.113} \ e^{-\frac{U+15.1}{32.95}} \end{aligned}$$

for *H* and *U*, respectively.

**Fig. 3:**
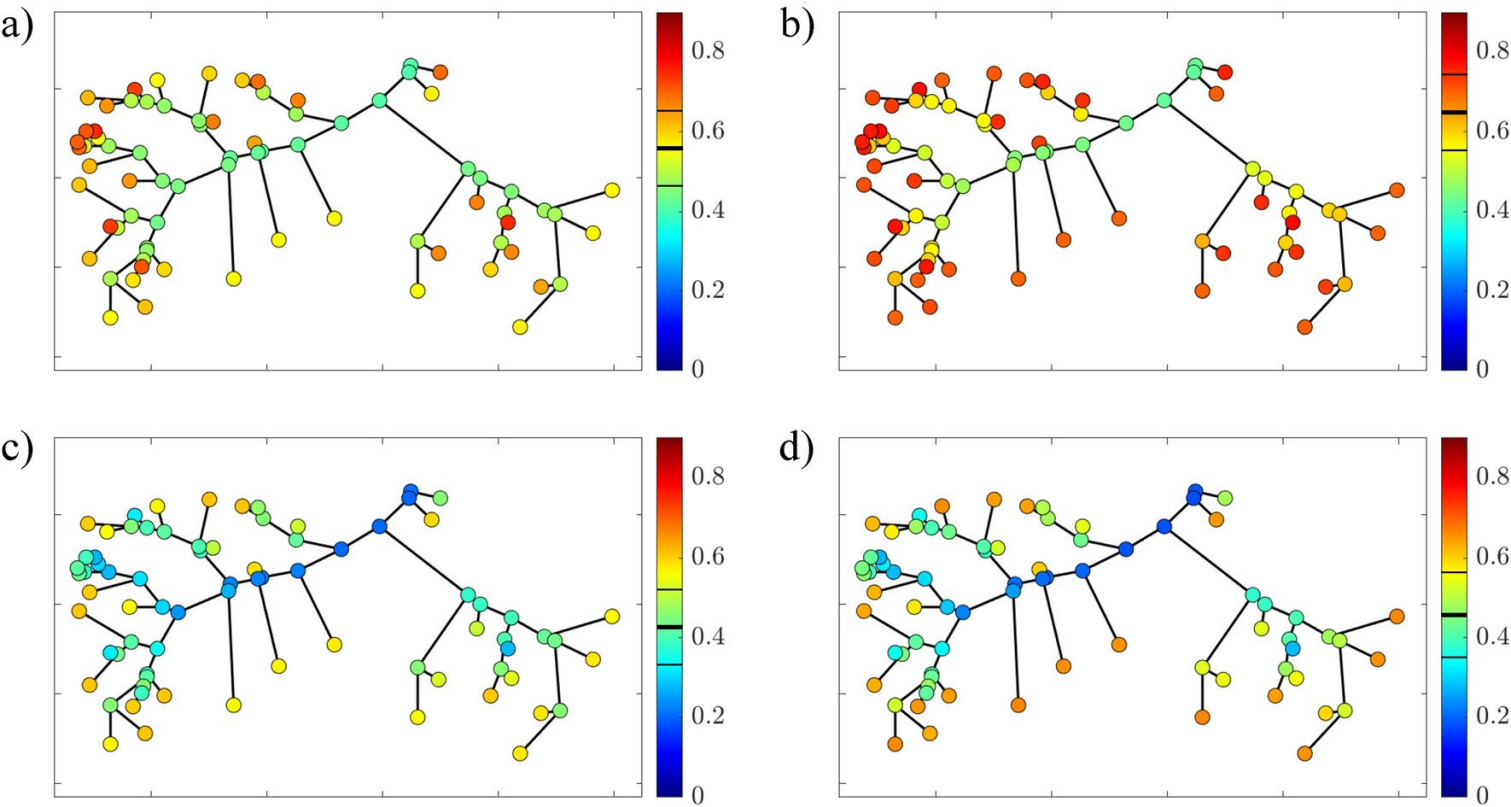
The compound HSI, HSI$_C$, for adults and juveniles according to two values of the flow discharge, $Q_0$. The thick and thin black lines on the color bar indicate the mean HSI$_C$ ± 1SD (standard deviation). (**a**) HSI$_C$ for adults and $Q_0$=0.2 m$^3$ s$^{-1}$; (**b**) HSI$_C$ for juveniles and $Q_0$=0.2 m$^3$ s$^{-1}$; (**c**) HSI$_C$ for adults and $Q_0$=0.6 m$^3$ s$^{-1}$; (**d**) HSI$_C$ for juveniles and $Q_0$=0.6 m$^3$ s$^{-1}$.

The obtained curves are used for sensitivity analysis of the HSI$_C$ with varying upstream flow discharge $Q_0$. As shown in Figure 3, small variations in $Q_0$ can lead to significant differences in the HSI$_C$. For instance, adult fish show a mean HSI$_C$ value decreasing from 0.55 (Figure 3a) to 0.44 (Figure 3c) when $Q_0$ increases from 0.2 to 0.6 m$^3$ s$^{-1}$, while for juveniles the HSI$_C$ is reduced from 0.64 to 0.46 for the same values of $Q_0$ (Figure 3b,d). The HSI dependence on the catchment hydrology makes the coupling of hydrodynamic and habitat models paramount in the context of river restoration planning and hydro-power exploitation. Habitat quality is based on values of flow velocity, *U*, and water depth, *H*, and in turn *U* and *H* are dependent on the flow discharge *Q*. Considering that river restoration or modified hydro-power exploitation may change the hydraulic characteristics at the reach scale, the set of Eq.s (7)-(10) may be useful to predict future habitat conditions of the restored river, and measure the project’s impact on it.

The proposed approach highlights the importance of coupling data on river hydraulics and habitat quality, as both factors play a major role in determining the abundance of fish in a certain area. In particular, hydrodynamics can vary significantly along a river network since rivers generally evolve from upstream, narrow, steep, and shallow cross-sections with high flow velocity to downstream wide, mild, and deep channels with low flow velocity. As a consequence, differences in water depth, *H*, and flow velocity, *U*, within the whole river network are reflected in the corresponding HSI values (Eq.s (7)-(10)). For the tested values of flow discharges (Figure 3), the HSI$_C$ for both adults and juveniles tend to show higher values in the marginal reaches (i.e., upstream reaches), rather than in the main channel.

On the scale of the entire river network, the HSI$_C$ for adults (Figure 3a,c) is lower than the one for juveniles (Figure 3b,d). Additionally, when $Q_0$ increases, the mean HSI$_C$ for the entire network decreases more for juveniles than for adults, with a more significant increase in standard deviation. As a consequence, younger individuals seem to be more sensitive to changes in the hydrological characteristics of the catchment.

**Fig. 4:**
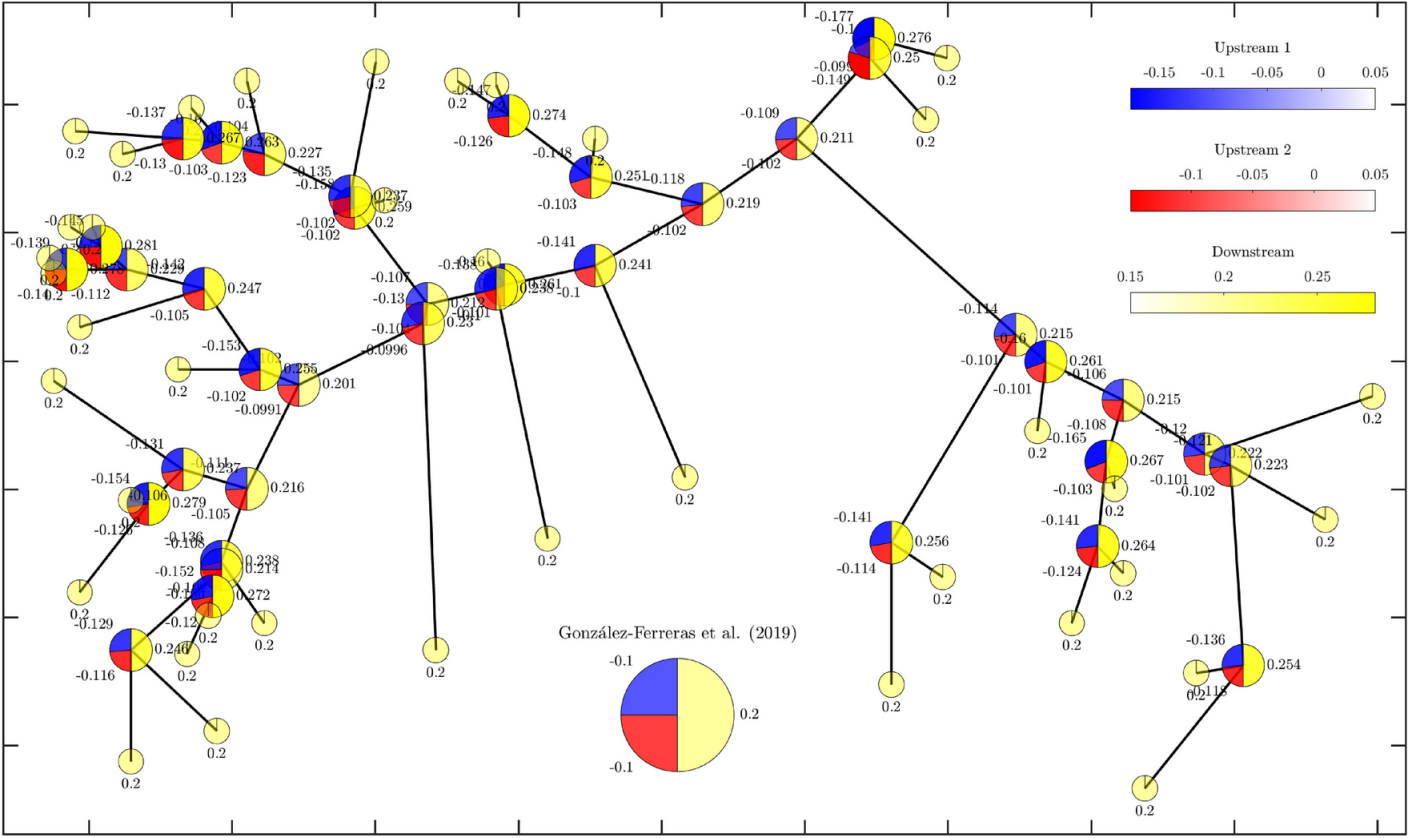
The bias for the upstream and downstream movement of adult fish as a function of the ratio between HSI (Eq.(3)) for $Q_0$=0.2 m$^3$ s$^{-1}$. The constant values from Gonz$\acute{\text {a}}$lez-Ferreras et al.^22^ are also shown.

As demonstrated above, the HSI for individuals of different age classes can significantly vary, not only spatially within the catchment, but also locally with time (e.g., daily, monthly, or seasonally). Therefore, modeling fish movement should account for temporal and spatial variations of catchment characteristics in terms of hydraulic properties. In the metapopulation model of Gonzales-Ferreras et al.^22^, the value of the bias parameter, *b*, defined as the difference in probability for an individual to move downstream and upstream, is the same for juveniles and adults, just with opposite signs. Their choice indicates the willingness to account for different trends in the movement direction (i.e. juveniles generally move upstream^19,20,29^, while adults move downstream^21,28^). Conversely, our proposed methodology weights *b* with the ratio between the HSI of the adjacent upstream and current nodes (Eq. (3)) and highlights the importance of considering habitat quality to determine the movement bias. As shown in Figure 4, by applying Eq. (3) with $Q_0$=0.2 m$^3$ s$^{-1}$, the *b* for the adults’ upstream movement, considering both upstream nodes, ranges between -0.067 and -0.176, which corresponds to a deviation from the original value (i.e., $b_0$=-0.1^22^) of -33% to +76%. For the sake of clarity, the source nodes show only downstream bias (yellow color in the pie chart in Figure 4), due to the absence of upstream branches, by definition of the river network.

## Discussion

In this work, we use a simple 1D hydrodynamic model to assess habitat suitability and the related fish movement bias, explicitly linking them to the hydraulic conditions of river reaches. The hydrodynamic model assumes a simple rectangular cross-section to calculate hydraulic properties such as water depth and mean flow velocity, due to the absence of geometrical measurements of all the cross-sections along the river network. If the cross-sectional shapes are known, the hypothesis of rectangular geometry can be relaxed, and the hydraulic properties can be calculated accordingly. Furthermore, Eq. (1) is based on the assumption of normal flow conditions, which are uncommon in real rivers due to the presence of in-channel structures (e.g., weirs), large-scale bedforms^31^, cross-sectional alterations (e.g., widening and narrowing), and other factors affecting flow surface elevation (e.g., vegetation-induced roughness and its spatial and temporal variation^32,33^). Nevertheless, these assumptions are quite straightforward since the data used for the hydraulic model are both reach- and monthly-averaged. Additionally, the hydrodynamic simulation is performed over a large spatial domain (i.e., catchment scale); thus a certain degree of hydraulic simplification is inherently required to ensure computational efficiency and avoid overfitting.

As last, we recall that the main objective of the simplified hydrodynamic tool is to estimate the hydraulic characteristics at the reach scale, within the whole river network, rather than to perform a detailed simulation of the local hydrodynamic conditions. In the latter case, more appropriate 2D hydrodynamic models should be considered (e.g., Basement^34^, Delft3D^35^).

**Fig. 5:**
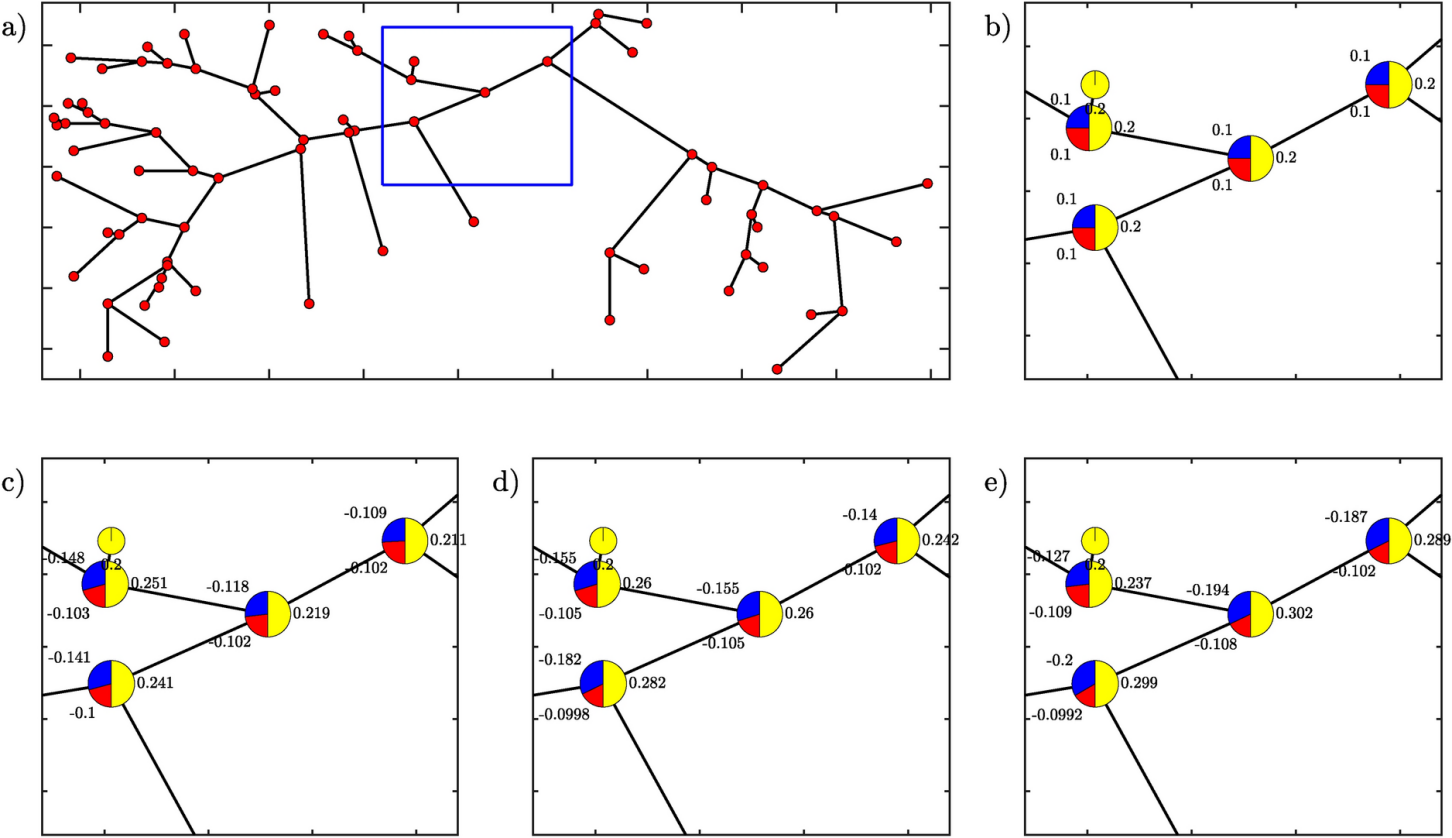
The movement bias of adult fish in the original model^22^ and the proposed framework (Eq. (3)-(4)), for different values of the flow discharge $Q_0$. In the pie charts, blue and red refer to the two upstream branches, and yellow refers to the downstream reach. (**a**) The modeled river network. Blue square shows the position of nodes in the next panels; (**b**) The constant movement bias in the original model^22^; The movement bias in the proposed framework for (**c**) $Q_0$=0.2 m$^3$ s$^{-1}$; $Q_0$=0.4 m$^3$ s$^{-1}$; $Q_0$=0.6 m$^3$ s$^{-1}$.

However, 2D simulations require significant efforts for calibration and runs; as such, 2D numerical tools are quite seldom used to model an entire river network. In this regard, numerical models integrating 2D hydrodynamic simulations have been developed to simulate habitat quality at the cross-sectional or reach scale, only (e.g., River2D^36^). Nevertheless, the use of a more refined hydraulic model does not ensure the capture of the local flow properties to which fish respond.

Conversely, 1D flow simulations offer both computational efficiency and the accuracy required for modeling habitat quality^37^. For that reason, 1D hydraulic tools have already been integrated into habitat quality models (e.g., PHABSIM^38^), in comparison to previously available frameworks (e.g., MesoHABSIM^39^). The proposed 1D hydrodynamic tool is robust enough to model average flow properties at the reach scale, yet some improvements may be further developed. For instance, the potential implementation of a more accurate description of the cross-sectional geometry has already been mentioned. Additionally, in Eq. (1) the reach-averaged bed slope can be replaced by the energy slope (i.e., permanent flow, instead of normal flow conditions) for all river reaches.

As far as metapopulation models are concerned, the integration of hydrodynamic tools is still in its infancy. In Gonz$\acute{\text {a}}$lez-Ferreras et al.’s model^22^, the population dynamics is modeled based on two flow conditions only (i.e., high flow and low flow), alternating throughout the year. Proper flow discharge values are not considered; therefore, computation of hydraulic characteristics is not explicitly required. In comparison to the model mentioned above, the proposed framework includes a hydrodynamic tool capable of capturing the main hydraulic properties driving the habitat characteristics of river reaches, with the additional benefit of computational efficiency and reliability.

We fit the Habitat Suitability Curves according to the database of Padoan et al.^6^ by testing several functions. Although other relationships may be derived, the proposed equations perform well within the observed ranges of water depth, *H*, and mean flow velocity, *U* (Figure 2c,d). Some displacements can be observed for very high mean flow velocities ($U>80$ cm/s, Figure 2d), likely due to the limited availability of data in this velocity range^6^. However, it is important to highlight that the habitat conditions in this region of the HSI curve are very poor^6,39,40^, even though the fitted relationships tend to overestimate the actual data values for both adults and juveniles (red and blue curves in figure 2c, respectively). In this case, avoiding excessively small values of HSI is preferable, as they could affect the computation of the bias (Eq. (3)).

The originality in the proposed methodology stands on the fact that it combines the habitat quality index with the modeling of fish movement and migration within the river network. Particularly, it defines a relationship between movement bias and habitat suitability, so that the latter acts as a scaling factor for the bias itself. The bias calculation accounts for the age class while considering the tendency of individuals to move downstream (adult fish^21,28^) or upstream (juveniles^19,20,29^). A logistic-type function is used for the bias-HSI relationship (Eq. (3)) since such relationships commonly emerge in natural processes involving biotic communities^41^, including riparian vegetation^42,43^, and specifically in fish-related processes^44,45^. Furthermore, in Eq. (3) and the value of $b_\text {min}$, we assume that individuals are still biased to move in the preferential direction, regardless of potentially poor habitat conditions in the destination reach.

Figure 5 compares the movement bias between the proposed framework and the model from the literature^22^. As in Figure 4, some nodes (i.e., the source nodes) show only downstream bias values. Notably, the movement bias changes not only according to the HSI$_C$-ratio between consecutive nodes but also with the values that are affected by flow conditions (compare panels c,d and e in figure 5). This finding is particularly relevant as it stresses the importance of spatial and temporal flow variability which is typically guaranteed by the natural flow regime^46^ or dynamic flow releases^47^. Conversely, constant values (Figure 5b) remain unaffected either by habitat, or flow conditions. In the case of Figure 5, changes in *b* are even more marked when the analysis is performed with different discharges (Figure 5c,d,e). When the discharge $Q_0$ increases from $Q_0$=0.2 m$^3$ to $Q_0$=0.6 m$^3$, the upstream *b* ranges from -0.021 and -0.2. These values, ranging from -79% to +100% with respect to the initial *b*, are reached in the simulation with the highest $Q_0$=0.6 m$^3$ (red and blue colors in Figure 5e).

An additional remark on improving the proposed framework is in order here. Future enhancements should facilitate its implementation in metapopulation models for network-scale ecohydraulic applications. A key step should be developing a compound Usable Area index able to represent the morphological and hydraulic variability of the local reach while avoiding time-consuming 2D hydrodynamic simulations. One possible approach is by linking the HSI to the index of hydro-morphological variability developed by Gostner et al.^48^ while also incorporating additional effects of flow inhomogeneities in terms of Suitable Area^6,49^. This will allow for fast calculations and will introduce a natural link with flow variability, whose functions are fundamental attributes of healthy alluvial river systems^12^. Moreover, the proposed framework applies to brown trout, but it can be readily adapted to other fish species and river systems, broadening its applicability and contribution to expanding the comprehensive population dynamic modeling.

A key aspect regards the validation of results, which is crucial to ensure reliable simulations of real-world conditions, particularly in complex natural environments. However, measurements regarding fish movement and population dynamics in river networks are rarely available in the literature^22^. Therefore, research efforts should aim at collecting on-site monitoring data.

## Conclusions

In this work, we propose a novel framework coupling a simple 1D hydrodynamic tool with data-derived Habitat Suitability Curves for juvenile and adult individuals of brown trout to compute the ecohydraulic-driven movement bias when fish migrate between nodes in a river network. The analysis highlights the influence of hydraulic properties in terms of flow discharge and habitat conditions, along with the fish properties (i.e., age class), on the movement bias of adult and juvenile brown trout. In particular, the results emphasize the tendency of fish to move towards areas with better habitat conditions (i.e., higher HSI) when compared to constant bias models. Even in its current form, and especially when integrated into metapopulation models, the proposed framework may assess the potential effects of reach-scale restoration projects on fish population dynamics by comparing pre-, and post-restoration conditions. Such assessment will help determine whether restoration measures positively or negatively impact the target species, and identify the optimal locations for the restoration efforts.

This study offers valuable insights into ecohydraulics by emphasizing the critical role of habitat quality in movement dynamics and metapopulation modeling. The proposed framework has significant potential for application in river restoration and management. By evaluating changes in the Habitat Suitability Index between pre- and post-restoration scenarios, this approach can help experts identify the most suitable locations for restoration projects. Furthermore, the ability to assess the outcomes of these measures would facilitate the recreation of habitats that more closely resemble their natural state, mitigating the effects of human impact. In addition, long-term simulations are essential to address the effects of climate change on restored rivers, thus enabling the development of more resilient management strategies.

## Data Availability

The data used in this work, as well as additional values for temperatures and substrate characteristics, can be found at https://doi.org/10.5281/zenodo.14639548.
